# Clinical study of the diagnosis of thyroid tumours using Raman spectroscopy

**DOI:** 10.1016/j.bjorl.2025.101568

**Published:** 2025-02-28

**Authors:** Qingjian He, Lianjin Qin, Yongqiang Yao, WenJuan Wang

**Affiliations:** a The First People’s Hospital of Huzhou City, Department of Breast and Thyroid Surgery, Huzhou, China; b Zhong Shan Hospital of Dalian University, Department of Breast and Thyroid Surgery, Dalian, Liaoning, China; c First People’s Hospital of Huzhou City, Department of Cardiovascular Diagnosis and Treatment Center, Huzhou, China

**Keywords:** Raman spectroscopy, Benign thyroid tumour, Thyroid malignancy, Nodular goitre, Papillary carcinoma

## Abstract

**Objective:**

The feasibility of the RS for the clinical diagnosis of thyroid tumours was explored.

**Methods:**

The tumour specimens from 30 benign patients and 30 malignant patients were collected. The collected specimens were subjected to RS and histopathological analysis. The Raman peak intensities of all the specimens were calculated, and the data were analysed using discriminant analysis.

**Results:**

(1) The prevalence rate of malignant tumours in females was as high as 76.7%. Central lymph node metastasis of malignant thyroid tumours accounted for 33.3% of cases, and lateral cervical lymph node metastasis accounted for only 6.7%. (2) The spectral intensity of malignant thyroid tumours was significantly greater than benign thyroid tumours at 1309 cm^−1^, which should be the characteristic peak of thyroid cancer. The accuracy, sensitivity, and specificity of the RS for differentiating benign from malignant thyroid tumours were 95%, 83.3% and 89.2%.

**Conclusion:**

RS is feasible for the diagnosis of thyroid tumours. This study provides experimental and clinical support for the wider application of RS in the evaluation of thyroid tissue.

**Levels of evidence:**

: Levels 4.

## Highlights

• RS provides unique spectral characteristics.
• RS diagnosis can be completed in a short time.
• Non-invasiveness, accuracy, rapidity, objectivity and cost-effectiveness.
• RS not cause any damage to tumour specimens, more objective and rapid.

## Introduction

For thyroid tumours with suspected malignancy, surgical resection is the best treatment, and pathological diagnosis by experienced pathologists is particularly important. However, the wait for pathological results during surgery is long, which greatly reduces surgical efficiency.^1^ Raman Spectroscopy (RS) is a non-destructive optical technique that relies on the inelastic scattering of photons derived from the vibrations of molecular bonds.^2^, ^3^, ^4^ When a photon interacts with a molecular bond, its frequency changes to produce a “Raman peak”.^5^, ^6^, ^7^ Different human tissues are composed of different proteins, nucleic acids, lipids and carbohydrates, and all tissues have a characteristic Raman spectra.^8^, ^9^, ^10^ Once human tissue becomes cancerous, the molecular configuration, conformation and composition ratio of the tissue change.^11^, ^12^ RS can detect these changes at the molecular level, and it is a diagnostic method with great potential.

RS is a highly sensitive analysis and imaging technology that is well tested in biomedical research, and it is primarily used in the diagnosis of various diseases, including cancer.^13^, ^14^, ^15^, ^16^ RS is widely used in the medical field, and it exhibits obvious advantages.^17^, ^18^, ^19^, ^20^, ^21^ (1) RS may be applied to solids, liquids, body fluids, soft tissues and other forms. (2) RS only needs the experimental sample and does not cause physiological damage to the sample. Other diagnostic methods require pre-treatment of the sample, which may cause different degrees of damage to the tissue sample itself. (3) RS technology may also be used for in vivo detection, which is the greatest advantage.

Shuming Nie used a portable Raman device called the “Spectro pen” to perform in vivo experiments on mouse mammary tumours and demonstrated that it accurately identified mammary tumour margins.^22^ RS was used to detect 20 patients with thyroid cancer and 18 patients with healthy thyroid tissues of different pathological types.^23^, ^24^ RS can be used to distinguish between normal and cancerous thyroid tissues with 100% sensitivity, specificity and accuracy and 93% sensitivity. There was 100% specificity and 95% accuracy in distinguishing between classical and follicular variants of papillary thyroid carcinoma. Recent studies show that RS is feasible for the diagnosis of thyroid tumours.^25^, ^26^ This study developed the label-free Surface-enhanced Raman spectroscop liquid biopsy combined with the CNN model to achieve a rapid and accurate diagnosis of thyroid cancer.^27^ Compared to other imaging methods, RS has the advantages of low background signals, high spatial resolution, high chemical specificity, multiplexing ability, good light stability and non-invasive detection ability. Raman imaging is a promising research tool in cancer diagnosis and provides molecular information to distinguish between cancerous and non-cancerous cells, tissues and body fluids.

The main purpose of this study was to determine the sensitivity and specificity of RS for the diagnosis of pathological properties of thyroid tumours to verify the feasibility of RS for the clinical diagnosis of thyroid tumours.

## Methods

### Statement of medical ethics

This study was performed in accordance with the Declaration of Helsinki (2013 edition).^28^ The procedures followed in this study and the collection of clinical data were approved by the Ethics Committee Board of Dalian University Zhongshan Hospital (licence number: 2019263). All patients or their relatives and healthy volunteers provided written informed consent.

### Specimen collection, grouping and storage

The following inclusion criteria were used: 1) Met the diagnostic criteria for primary thyroid tumours; 2) Met the collection conditions (tumour specimens ≥ 1 cm); and 3) Patients required surgical treatment. The following exclusion criteria were used: 1) Had other tumours; 2) Were pregnant; 3) Had mental disorders; or 4) Did not provide informed consent.

The data of 60 patients with thyroid tumours were collected from Zhongshan Hospital Affiliated affiliated with Dalian University: 30 patients had benign thyroid tumours, and 30 patients had malignant thyroid tumours.

According to the study of Xia *et al*, the accuracy rate of diagnosis of thyroid nodules by Raman spectroscopy was 93.65%.^28^ The equivalent sample size of the two groups was used, *δ* was set at 0.2, and the sample size ratio κ of the experimental group/control group was set at 1. The minimum sample size was calculated as 26 cases. In this experiment, 30 cases were selected to meet the requirements of minimum sample size.

Fresh thyroid tumour tissue samples were immediately placed into 1.5 mL frozen storage tubes and stored in liquid nitrogen for preservation. Frozen sections were created within 30 min after isolation, and paraffin sectioning was performed within 72 h.

The tumour specimens were fixed overnight in Bouin’s solution at 4 °C, rinsed with running water for 6 h then dehydrated. The specimens were treated with xylene and placed in a fresh paraffin solution overnight. Paraffin-coated and marked specimens were treated overnight. The thickness of the sections was set to 5 microns, and H&E staining was performed.

In order to ensure the preparation of research data, the size of tumour tissues for RS detection was labelled, and the size of tissue samples for each group was set to 5 × 5 mm, and the weight was guaranteed to be 20 mg.

### Experimental equipment

During operation of the RS, the laser was fitted to a microscope through a single-mode fibre and focused on the sample for testing on a motorised stage. The scattered Raman signal was passed through two filters and was captured by a CCD detector. The signals were recorded and reflected as the RS on a computer. The thyroid tumour specimens were placed in a Petri dish covered with PBS solution, and the tumours were dissected under a stereoscope with microsurgical tweezers. Two different locations for each thyroid tumour specimen were selected for RS scanning. The calibration value of the Raman spectrum signal in this experiment was 1445 cm^−1^, the selected laser wavelength was 532 nm, the power was 10 mW, and the single scan time was 10 s.

### Characteristic peak analysis

The RS data were automatically obtained using OPUS 6.5 software. To ensure that all RS were comparable, several data processing steps were performed prior to analysis: 1) Spectral calibration using the known spectra of silicon slices; 2) Correction of the spectral response of the system using a tungsten white light source diffusely scattered by a reflectance standard BaSO_4_; 3) Fluorescence background removal using fifth-order polynomial fitting;^29^ 4) Baseline correction using a stretched rubber band between the spectrum endpoints that follows the spectrum minima;^30^ and 5) Data normalisation by dividing each spectral point by the area of the total intensity of the spectrum. The RS signal of the tumour was represented by the average spectral peak of the two scanning sites on each tumour specimen. The average RS signals of benign tumours and malignant tumours were compared, and the locations of the spectral peaks were marked. Discriminant analysis was used to process the data, and the ability of RS to distinguish thyroid tumour properties was tested using the “leave one out cross-validation analysis”. Each spectral data point was detected using “leave one tissue out”, and the overall ability of RS to distinguish benign and malignant thyroid tumours was obtained, which was reflected in sensitivity and specificity.

Leave One Out Cross-Validation analysis (LOOCV) is a model validation method suitable for small data sets.^31^ The principle is to select one sample at a time from the data set as the test set and the rest as the training set.^32^ For example, a dataset of 100 samples is tested with 1 sample at a time, trained with 99 samples, and repeated 100 times. Finally, all test results are summarized and average performance indicators (such as accuracy, sensitivity, specificity, etc.) are calculated. In medical diagnosis, LOOCV ensures the model's performance on each individual by independently verifying each sample, improving diagnostic accuracy and reliability.

GC–MS first simulated missing values in the original data using a method of filling in one-half of the minimum value. To analyse the downstream data more accurately and remove interfering and complex data, the quartile range was used to filter the data, and the filtered data were standardised.

## Results

### Clinical data analysis

Table 1, Table 2 show that nodular goitre was the main pathological type of benign tumour in this experimental study, and papillary thyroid carcinoma was the main pathological type of thyroid cancer. Thyroid tumours were most common in women and accounted for 76.7% of malignant tumours. The size of primary tumours collected in this study was all greater than or equal to 1 cm, and the proportion of thyroid tumours with pT1 stage was 86.7%. Central lymph nodes were more common in patients with malignant thyroid tumours, and cervical lymph nodes were less common and accounted for only 6.7%.

**Table 1:** Clinical data of the patients.

Clinicopathological parameter	Number of cases (number)	Percentage (%)
Pathological type		
Nodular goitre	28	46.7%
Thyroid adenoma	2	3.3%
Thyroid carcinoma	30	50.0%
Age (years)		
≤40	36	60.0%
>40	24	40.0%
Sex		
Female	43	71.7%
Male	17	28.3%
Tumour Size (cm)		
≤2	46	76.7%
>2	14	23.3%

**Table 2:** Clinical data of patients with thyroid carcinoma.

Clinicopathological parameter	Number of cases (number)	Percentage (%)
Age (years)		
≥40	18	60%
<40	12	40%
Sex		
Female	23	76.7%
Male	7	23.3%
Pathological Type		
Papillary Carcinoma	30	100%
Tumour Size (T > 1 cm)		
pT1	26	86.7%
pT2	4	13.3%
Lymph node metastasis (N)		
N0	18	60%
N1a	10	33.3%
N1b	2	6.7%

### Morphological characteristics of thyroid tumours

**Fig. 1:**
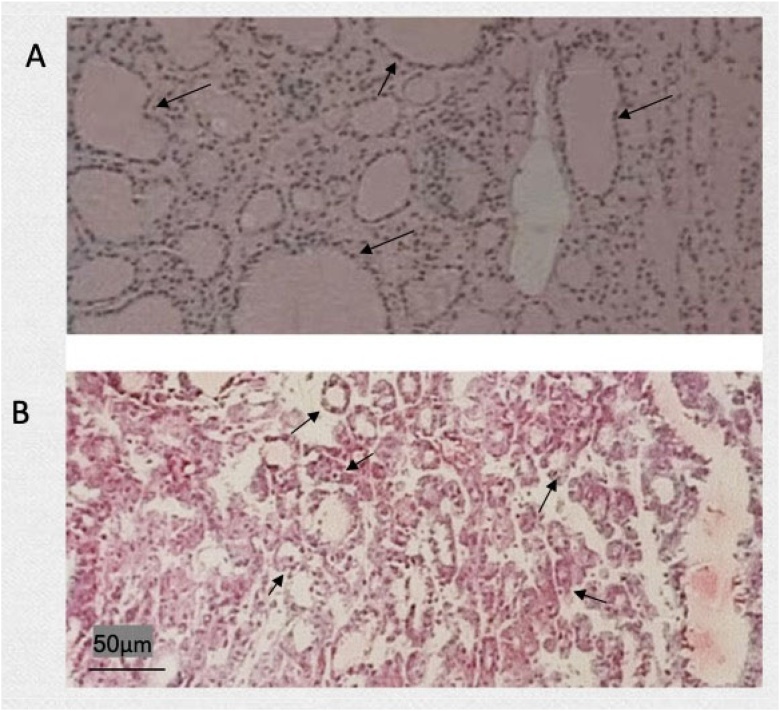
Frozen and paraffin sections of nodular goitres. (A) Rapid frozen section of a nodular goitre; (B) Paraffin section of a nodular goitre.

The gross surface of the nodular goitre was uneven and consisted of multiple nodules of different sizes, each of which had a clear boundary and no complete envelope. Most of tumours were accompanied by bleeding, necrosis, cystic changes, fibrosis, and calcification. As shown in Fig. 1, the microscopic features were diverse morphology, repeated follicular hyperplasia and degeneration. The follicles became larger, and the cavity was filled with colloidal substances.

**Fig. 2:**
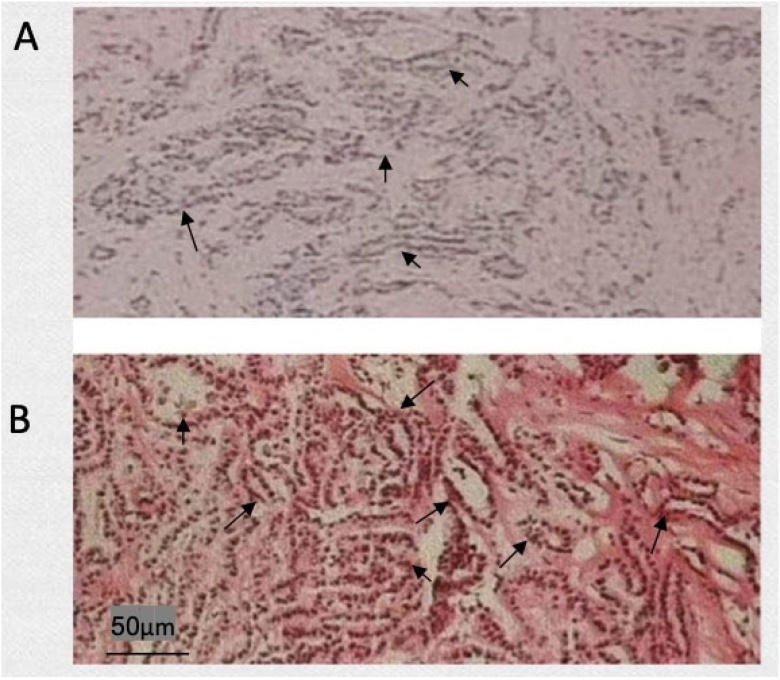
Frozen and paraffin sections of papillary carcinoma. (A) Rapid frozen section of papillary carcinoma; (B) A paraffin section of papillary carcinoma.

The macroscopic appearance of papillary thyroid carcinoma was mostly grey with white hard nodules without a complete envelope, and the tumour obviously spread to the surrounding thyroid parenchyma. As shown in Fig. 2, the solid part of the tumour accounted for approximately 50%‒70% of the tumour, with a complex, branchlike, disordered papillary structure, irregular cell nuclei, ground glass or pale, active mitosis, crowded cell nuclei, and some subtypes with a large number of sand bodies and dense lymphocyte infiltration.

### Raman spectrum test results

**Fig. 3:**
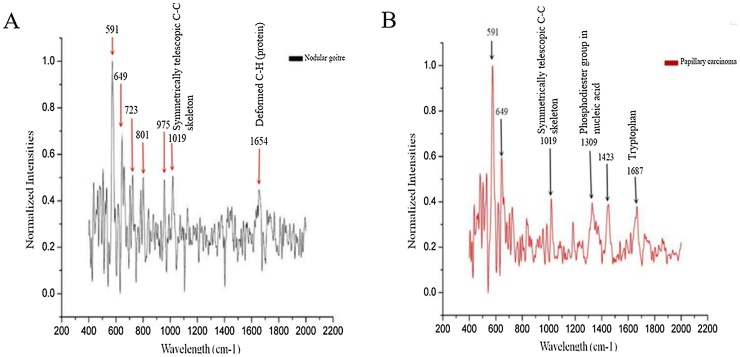
Homogenised Raman spectra of benign and malignant thyroid tumours. (A) Uniform Raman spectroscopy of nodular goitre; (B) Homogenised Raman spectroscopy of papillary carcinoma.

As shown in Fig. 3A, benign thyroid tumours showed spectral peaks at 591 cm^−1^, 649 cm^−1^, 723 cm^−1^, 801 cm^−1^, 975 cm^−1^, 1019 cm^−1^, and 1654 cm^−1^. As shown in Fig. 3B, malignant thyroid tumours showed spectral peaks at 591 cm^−1^,649 cm^−1^, 1019 cm^−1^, 1309 cm^−1^, 1423 cm^−1^, and 1687 cm^−1^.

**Fig. 4:**
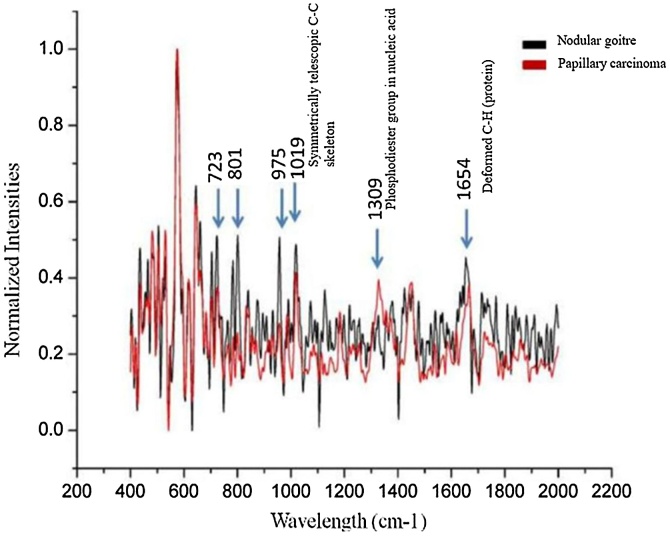
Comparison of homogenised Raman spectra between benign and malignant thyroid tumours.

As shown in Fig. 4, the Raman peak at 1309 cm^−1^ for malignant thyroid tumours was significantly different than benign tumours, and it should be considered the characteristic peak of thyroid cancer in this study. Rau *et al* identified 1006 cm^−1^, 1156 cm^−1^, and 1520 cm^−1^ as the characteristic peaks for the diagnosis of papillary thyroid cancer.^23^ The difference in experimental data at home and abroad may be related to differences in the sensitivity and data analysis system of the Raman spectrometer.

The rapid proliferation and faster metabolism of thyroid malignant tumour cells lead to changes in the secondary structure of proteins, and the higher content of nucleic acids in the tumour. The vibration patterns of these specific components may determine different peak signals. It has been suggested that differences in the biochemical composition of carbohydrates, nucleic acids, lipids and proteins between benign and thyroid cancer cell lines lead to differences in Raman peaks.^25^

As shown in Table 3, comparison of the homogenised RS of nodular stroma and papillary thyroid carcinoma revealed that the peak values of the RS overlapped at 1019 cm^−1^, and the corresponding substances is the symmetrically expanded C—C skeleton. Compared to tuberous goitres, papillary thyroid carcinomas differed in 1309 cm^−1^, 1423 cm^−1^ and 1687 cm^−1^. The main substance corresponding to the peak at 1309 cm^−1^ is the Phosphodiester group in nucleic acid. The main substance corresponding to the peak at 1687 cm^−1^ is tryptophan.

**Table 3:** Raman frequencies and their assignments.

The peak value of nodular goitre (cm^−1^)	Thyroid papillary carcinoma peak (cm^−1^)	Corresponding substance
1360	1360	Tryptophan
1204	1204	Amide Ш
1684		Amide I ν(C=O)
600		Nucleotide
1242−1266		Amide Ш collagen
856		Collagen
918		Proline, hydroxyproline acid
1019	1019	Symmetrically telescopic C—C skeleton
	1309	Phosphodiester group in nucleic acid
	1224	Amide Ш (β-sheet structure)
1654		Deformed C—H (protein)
	1687	Tryptophan

As shown in Table 4, RS had a strong ability to distinguish benign and malignant tumours by further cross-validation of the “leave one method” for the determination of benign and malignant tumours of the thyroid.

**Table 4:** “Leave-one spectrum out” cross-validation for the discrimination of benign thyroid tumours from malignant thyroid tumours.

Histological diagnosis	Algorithmic prediction	
	Benign thyroid tumour	Malignant thyroid tumour
Benign thyroid tumour	25	5
Malignant thyroid tumour	3	25

### Pathological identification results

As shown in Table 5, the pathological identification results of 60 tumour specimens by RS were compared with the intraoperative pathological diagnosis and postoperative paraffin pathological diagnosis results. The accuracy of the identification was 95% (57/60), the sensitivity was 83.3%, and the specificity was 89.2%. Confidence interval for sensitivity [0.66‒0.94], confidence interval for specificity [0.71‒0.97]. Thirty cases of benign thyroid tumours were identified by RS, but 2 cases of thyroid adenoma were not correctly identified. The accuracy rate was 93.3% (28/30). For papillary thyroid carcinoma, 29 of 30 cases were successfully identified, with an accuracy of 96.7% (29/30).

**Table 5:** Capacity of Raman spectroscopy for the discrimination of benign thyroid tumours from malignant thyroid tumours.

	Benign tumours/Malignant tumours
Sensitivity	25/30 = 83.3%
Specificity	25/28 = 89.2%

### Power analysis

The effect size of Raman spectral peaks for benign and malignant tumours is calculated to be 0.89, implying a large difference between the two groups. Assuming a significance level of 0.05 and a sample size of 30, the efficacy is about 0.87, suggesting that we have sufficient statistical power to detect a significant difference between the two groups under these conditions.

### Comparison of diagnostic times

As shown in Table 6, the average diagnostic time of RS was relatively fast. In this study, the average diagnostic times were 24.37 ± 0.46 s for benign tumours, and 26.98 ± 0.55 s for benign tumours.

**Table 6:** Average diagnostic times of Raman spectroscopy.

Diagnostic mode	The average diagnosis of benign tumours (s)	The average diagnosis of malignant tumours (s)
RS detection	24.37 ± 0.46	26.98 ± 0.55

### Study site

Our study was conducted at Zhongshan Hospital Affiliated to Dalian University.

### Ethics committee

The Ethics Committee Board of Dalian University Zhongshan Hospital (licence number: 2019263).

## Discussion

The Raman scattering technique has become one of the most widely used spectral and imaging techniques in tumour nanomedicine due to its high spatial resolution, high chemical specificity and multiple modes.^33^, ^34^, ^35^, ^36^ Our results showed that the combination of histology and Raman microscopy clearly integrated morphological and biochemical observations to greatly improve the efficiency and reliability of the differential diagnosis of benign and malignant thyroid nodules. These results pave the way for the integration of tumour diagnostic mechanisms and the development of new treatment strategies.

RS and Surface-Enhanced Raman Scattering (SERS) improve the detection and monitoring of various diseases, especially cancer, with or without support by multifunctional active nanosystems.^37^, ^38^, ^39^ Once human tissue cells become cancerous, amino acids, lipids and other substances change, and RS peaks with different characteristics are formed. Therefore, RS may be used to diagnose diseases at the molecular and cellular levels.^40^, ^41^, ^42^, ^43^ For example, biological substances, such as cells, tissues and DNA/RNA, may be directly detected.^44^, ^45^, ^46^ The results of this study promote the development of Raman optical technology for the clinical diagnosis of tumour properties.

The incidence of thyroid cancer is increasing globally, and this increase is almost entirely due to an increase in papillary thyroid cancer.^47^, ^48^ RS provides insight into tissue status via the assessment of chemical composition and distinguishes diseases based on spectral features. We demonstrated the feasibility and repeatability of RS technology. The identified characteristic peak of thyroid cancer and the cross-verified sensitivity and specificity were 83.3% and 89.2%, respectively. Puppel *et al* studied carotenoids located in human lymphocyte subsets and natural killer cells and demonstrated that the concentration of carotenoids in CD4^+^ lymphocytes was very high.^49^ Some scholars proposed the use of carotenoids as Raman biomarkers in breast cancer pathology.^50^, ^51^ Carotenoids primarily exist in the cellular region of thyroid papillary carcinoma, and this substance may be used as the Raman characteristic substance of thyroid papillary carcinoma. The levels of α-carotene, β-carotene, γ-carotene and other substances are significantly different between papillary thyroid carcinoma and nodular thyroid gland.^52^, ^53^, ^54^

Thyrotropin-Releasing Hormone (TRH) is a judgement index for evaluating the therapeutic effect of hyperthyroidism and hypothyroidism. The development of RS to detect thyrotropin and evaluation of its sensitivity and specificity are highly important. The Study have shown that developed a Surface-Enhanced Raman Spectroscopy (SERS) sandwich immunoassay, which combined a dimethyl amino-azobenzene molecule with a thyroid stimulating hormone antibody via covalent bond binding and detected a small concentration of thyroid stimulating hormone.^55^ The Study have shown that RS distinguished a group with normal thyroid function from a group with total thyroidectomy with an accuracy of 100%.^56^ Current data and suggest that spectral evaluation has predictive value in identifying characteristics associated with thyroid hormone-related progression.

Although RS distinguished papillary thyroid carcinoma from nodular goitre in vitro, there are many problems to solved before its final clinical application. 1) The safety of laser RS requires further rigorous research. 2) Another shortcoming is the lack of normal thyroid tissue in the current study, and the RS peak of normal thyroid tissue was not verified. The lack of normal thyroid tissue is not conducive to the establishment of a more complete and accurate model database, limits the universality of study results, and may reduce the feasibility of Raman spectroscopy for clinical diagnosis. 3) Samples of other pathological types, such as follicular carcinoma and medullary carcinoma, were not collected in this study, and large amounts of data to support the identification of pathological types of thyroid malignancies are lacking. There are many specific types of papillary thyroid carcinoma, such as cell type, diffuse follicular type, columnar cell type, and diffuse sclerotic type. Whether the Raman peaks of these different types of papillary carcinoma are consistent needs further study. 4) The tumour specimens used in this study were cryopreserved. The freezing of tissues may potentially cause certain interference to the experimental results. Although cryopreservation can have some effect on Raman spectral signals, this effect can be significantly reduced by optimizing cryopreservation conditions, thus ensuring that cryopreservation thyroid tissue can still be used for high-quality Raman spectroscopy. These optimizations include the use of cryoprotectants (glycerin) and the control of freezing rates, which are important for improving the practicality and accuracy of Raman spectroscopy in clinical applications.^57^

Shuming Nie used a portable Raman device called the “Spectro pen” to perform in vivo experiments on mouse mammary tumours and demonstrated that it accurately identified mammary tumour margins.^22^ Certain study invented a Raman endoscopy system that used a 1.8 mm Raman probe device in the endoscope to perform real-time scanning during surgery.^58^ If the Raman endoscope system can be applied for pathological diagnosis of the tumour margin in breast-conserving surgery for breast cancer, it will greatly improve the safety and efficiency of surgery.

RS has certain advantages over ultrasound-guided needle biopsy in the diagnosis of thyroid tumours. First, RS provides unique spectral characteristics by detecting the molecular vibrations of biological tissues, which is helpful for distinguishing the types of thyroid tumours. Although ultrasound-guided needle biopsy provides tissue samples for pathological examination, there may be deviations or insufficient sample acquisition in some cases, which may lead to false-negative or false-positive results. Second, RS diagnosis can be completed in a short time and reduce the waiting time for results. The steps of rapid freeze pathology diagnosis include specimen preparation (fixation and freezing, 10–20 min), section (frozen section and staining, 10–20 min), microscopy (assessment of cell structure and morphology, 15–30 min), and report writing (communication of preliminary results, 5–10 min). From the time the specimen is sent to the pathology department to the issuance of a preliminary report, it usually takes 30 min to 1 h. Ultrasound-guided needle biopsy generally requires time for sampling, processing, and pathological examination, and the results are difficult to obtain. In conclusion, RS has the advantages of non-invasiveness, accuracy, rapidity, objectivity, and cost-effectiveness in the diagnosis of thyroid tumours compared to ultrasound-guided needle biopsy.

The numerous experimental studies support the potential of RS for cancer diagnosis.^59^, ^60^, ^61^, ^62^ In addition to the problems encountered in the current experiment, the entire RS analysis system has some additional problems that must be improved. 1) The sensitivity of the instrument must be improved. Sensitivity reflects the direct ability of RS to evaluate tumour properties, and sensitivity is the most important issue. The difference between the Raman peaks of tumour tissue and normal tissue is not obvious in some studies, and the difference between the Raman peaks of different individuals is not obvious, which directly affects the feasibility of the clinical application of this system. 2) The data analysis system is cumbersome. Although RS can diagnose the nature of tumours faster and more directly than subjective pathological diagnosis, the subsequent data calculation and analysis are very tedious. 3) Whether the technology can be widely used in the clinic also needs the support of optical experts, who must know more about clinical needs to continuously improve the technology.

Our experimental results preliminarily confirmed that RS may be a useful tool for the diagnosis of thyroid tumours. The experimental results also provide data and theoretical support for the application of RS in the diagnosis of thyroid cancer. The obtained results also demonstrate the great potential of RS to support histopathological evaluation and improve the reliability of cancer diagnosis. Compared to existing pathological diagnosis techniques, the greatest advantage of RS is that it is faster. We should focus on this technology to evaluate other pathological factors of thyroid disease, improve the sensitivity and specificity of this technology, and improve its clinical application.

In future studies, we will include more comprehensive tissue samples for comparison to strengthen the study. Multi-center studies can be conducted to explore Raman spectroscopy in different populations or different living environments to detect differences, which will help to verify the diagnostic accuracy of this technology and the feasibility of clinical pathologic diagnosis.

## Conclusion

RS may be used in the clinical diagnosis of thyroid tumours. The diagnosis time is very fast and does not cause any contact damage to tumour specimens, which can provide a more objective and rapid basis for the diagnosis of thyroid tumours.

## Funding

This study was funded by the Huzhou Science Bureau of Zhejiang Province (2021GYB04).

## Conflicts of interest

The authors declare no conflicts of interest.
